# A Novel *AICDA* Splice-Site Mutation in Two Siblings with HIGM2 Permits Somatic Hypermutation but Abrogates Mutational Targeting

**DOI:** 10.1007/s10875-022-01233-5

**Published:** 2022-03-05

**Authors:** Johannes Dirks, Gabriele Haase, Tineke Cantaert, Lea Frey, Moritz Klaas, Christian H. Rickert, Hermann Girschick, Eric Meffre, Henner Morbach

**Affiliations:** 1 Pediatric Immunology, University Childrens’ Hospital Würzburg, Würzburg, Germany; 2 Immunology Unit, Institut Pasteur du Cambodge, Phnom Penh, Cambodia; 3 Institute of Pathology, Würzburg University, Würzburg, Germany; 4 Pediatric Rheumatology, Vivantes Hospital Friedrichshain, Berlin, Germany; 5 Department of Pathology, Vivantes Hospital Friedrichshain, Berlin, Germany; 6 German Center for Growth and Development “DEUZWEG”, Berlin, Germany; 7 Department of Immunobiology, Yale University School of Medicine, New Haven, CT USA; 8 Center for Rare Diseases – Reference Center Northern Bavaria (ZESE), Würzburg, Germany

**Keywords:** Hyper-IgM syndrome type 2, *AICDA*, AID-ΔE4a, AD-AID, Mutational targeting, Somatic hypermutation

## Abstract

Hyper-IgM syndrome type 2 (HIGM2) is a B cell intrinsic primary immunodeficiency caused by mutations in *AICDA* encoding activation-induced cytidine deaminase (AID) which impair immunoglobulin class switch recombination (CSR) and somatic hypermutation (SHM). Whereas autosomal-recessive AID-deficiency (AR-AID) affects both CSR and SHM, the autosomal-dominant form (AD-AID) due to C-terminal heterozygous variants completely abolishes CSR but only partially affects SHM. AR-AID patients display enhanced germinal center (GC) reactions and autoimmune manifestations, which are not present in AD-AID, suggesting that SHM but not CSR regulates GC reactions and peripheral B cell tolerance. Herein, we describe two siblings with HIGM2 due to a novel homozygous *AICDA* mutation (c.428-1G > T) which disrupts the splice acceptor site of exon 4 and results in the sole expression of a truncated AID variant that lacks 10 highly conserved amino acids encoded by exon 4 (AID-ΔE4a). AID-ΔE4a patients suffered from defective CSR and enhanced GC reactions and were therefore indistinguishable from other AR-AID patients. However, the AID-ΔE4a variant only partially affected SHM as observed in AD-AID patients. In addition, AID-ΔE4a but not AD-AID patients revealed impaired targeting of mutational hotspot motives and distorted mutational patterns. Hence, qualitative defects in AID function and altered SHM rather than global decreased SHM activity may account for the disease phenotype in these patients.

**Supplementary Information:**

The online version contains supplementary material available at 10.1007/s10875-022-01233-5.

## Introduction

AID-deficiency results from deleterious mutations in *AICDA* encoding activation-induced cytidine deaminase (AID) and causes hyper-IgM syndrome type 2 (HIGM2) in humans [1]. AID is essential for initiating class-switch recombination (CSR) and somatic hypermutation (SHM) of immunoglobulin genes in B cells [2]. Hence, defects in AID result in abolished CSR and inadequate generation of the antibody isotypes IgG, IgA and IgE. In addition, affinity maturation of antibodies is impaired due to the lack of SHM in immunoglobulin genes [3].

On a molecular level, AID deaminates deoxycytidine (dC) to deoxyuridine (dU) in distinct motives within the variable (V) or switch (S) regions of immunoglobulin genes, thereby initiating SHM or CSR, respectively [4]. In SHM, AID particularly targets WRCY/RGYW hotspot motives in V regions (where R = purine, Y = pyrimidine, and W = A or T) [5]. Additionally, the S region targeted in CSR is enriched in the AGCT sequence, which is a palindromic version of the above-mentioned SHM hotspot motives. During CSR, deamination at both strands is followed by the removal of dU by either the uracil DNA-glycosylase (UNG) or components of the mismatch repair (MMR) pathway leading to double-strand breaks (DSB) and subsequently joining of the VDJ-segment with α, γ or ε constant region exons [6]. During SHM, following the initial deamination several downstream error-prone DNA-repair pathways may be engaged that further diversify the mutational pattern. Up to now, five different molecular pathways are known that process AID-initiated dU in V regions and differentially operate on the pattern of SHM [6, 7].

Most patients with AID-deficiency carry homozygous or compound-heterozygous *AICDA* mutations, which are inherited as an autosomal-recessive trait (AR) and affect both CSR and SHM [8]. Most of these variants are complete loss-of-expression and/or –function and mainly affect the nuclear localization signal or the cytidine deaminase domain itself. However, some patients only carry heterozygous *AICDA* mutations, which transmit the disease in an autosomal-dominant (AD) pattern. These distinct AD mutations (e.g., V186X and R190X) truncate the C-terminal nuclear export signal of AID, which result in an accumulation in the nucleus of mutated AID proteins that are unable to promote CSR but only partially affect SHM [9–12]. Hence, other mechanisms than abolished enzymatic activity must account for defective CSR in these patients, and involvement of several co-factors interacting with AID has been indicated by these observations [11, 13, 14].

AID-deficient patients suffer from recurrent infections most often affecting the respiratory tract [15]. Furthermore, autoimmune manifestations and/or lymphoproliferation may develop in AR-AID but not AD-AID patients, which both display defects in the peripheral B cell tolerance checkpoint [16, 17]. However, B cell tolerance is further breached in AR-AID patients as evidenced by exaggerated germinal center (GC) reactions, increased T follicular helper (T_FH_) cells and secretion of antinuclear antibodies (ANAs). In addition, patients with UNG-deficiency, which impairs CSR but not SHM, did not display broken B cell tolerance nor enhanced GC reactions, suggesting that SHM but not CSR regulates both features in humans [16].

Herein, we further refine this observation by describing two siblings with HIGM2 due to a novel homozygous *AICDA* mutation (AID-ΔE4a) that leads to the production of truncated AID proteins and results in impaired CSR but selectively impinges on SHM targeting, suggesting a rather qualitative impairment of SHM in these patients.

## Material and Methods

### Sample Preparation, Flow Cytometry and Cell Culture

Peripheral blood mononuclear cells (PBMCs) were purified with Ficoll density gradient centrifugation. For flow cytometry or cell sorting, PBMCs were stained in 1X PBS 0.5% BSA with appropriate antibodies at 4 °C for 30 min. Flow cytometry data were acquired on a FACSCanto II (BD Biosciences) and analyzed with FlowJo version 10 (Tree Star). For assessing *in vitro* immunoglobulin CSR, CD19^+^CD27^−^IgM^+^ naïve B cells were sorted on a FACSAria III (BD Biosciences) and stimulated with CD40L (5 µg/mL; Biolegend) and IL-21 (100 ng/mL; Preprotech). After 5 days, expression of IgG and IgA was assessed on CD19^+^CD27^+ ^B cells using flow cytometry.

### Immunohistochemistry

Excised tonsil and adenoid tissues were fixed in 4% paraformaldehyde and embedded in paraffin after dehydration in alcohol. Immunohistochemistry was performed using standard protocols.

### DNA Sequencing

Whole-exome sequencing (WES) was performed by a commercial provider using the SureSelect Human All Exon 50 Mb kit on a Illumina HiSeq 2500 system followed by an in-house analysis work-flow (CeGaT, Tübingen, Germany).

Sanger sequencing: The following primers were used for the amplification (AID Exon 4/5 FW CCCCGAGGAAATGAGAAAAT, AID Exon 4/5 REV GCAGAGATATTTCATCGTGTGTG) and sequencing (AID Ex4 REV Seq AGAGGGCTCTGAATGGTGAAAC) of the *AICDA* intron 3/exon 4 junction. PCR was carried out on genomic DNA obtained from PBMCs using GoTaq DNA Polymerase (Madison, WI, USA). For the analysis of exon/intron splicing, RNA obtained from CD40L/IL-21 stimulated naïve B cells was reverse-transcribed using iScript cDNA synthesis kit (Bio-Rad, Hercules, CA, USA). The following primers were used in RT-PCR for the amplification of the exon 3/4 junction of *AICDA*: FW TACTTCTGTGAGGACCGCAA, REV CATACAGGGGCAAAAGGATG. Sanger sequencing of purified PCR products was performed by a commercial provider (MWG eurofins, Martinsried, Germany).

### Single-Cell PCR and Immunoglobulin Repertoire Sequencing

Single CD19^+^CD27^+^IgM^+^ non-switched memory (NSM) B cells were sorted on a FACSAria III into 96-well PCR plates and immediately frozen on dry ice. RT-PCR amplification and sequencing of the variable and µ constant region of the immunoglobulin heavy chain (IgH) gene were performed as described before [16, 18]. IgH sequences derived from NSM B cells of previously described patients (AD-AID-12, AD-AID-13 and AD-AID-18 as AD-AID patients, AID01, AID04, AID05, AID06 and AID17 as AR-AID patients [16, 19]) were obtained using the same experimental approach and served as disease controls.

### Immunoglobulin Repertoire Analysis

After manual quality control of sequencing chromatograms, IgH sequences were aligned to germline sequences and processed using IMGT/HighV-QUEST with standard settings [20]. The output files were further analyzed using ARGalaxy [21]: Sequences covering the complete IgH sequence from CDR1 to CDR3 were assessed for SHM and associated hotspot motives. The location, predicted mutation pathways and effect on amino acid sequence were assessed for each mutation, and the mutational frequency was calculated for each IgH sequence. Corrected mutation numbers and frequencies as well as corrected A over T ratios were computed as described [7].

### Statistical Analysis

Using GraphPad Prism® 9, unpaired two-sided Student’s t-test was applied for analysis of continuous variables. Depending on the sample size, Fisher’s exact t-test or Chi-square with Yates’ correction was used for categorical variables.

## Results

### Clinical and Laboratory Findings

**Fig. 1:**
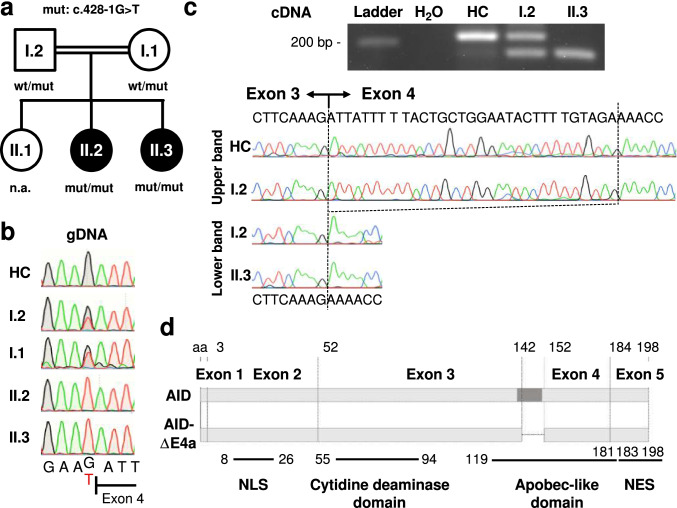
Identification of a homozygous AID-ΔE4a splice site variant in two patients with Hyper-IgM syndrome. **a** Pedigree of the affected sisters with clinical phenotype of hyper-IgM syndrome (II.2 and II.3). Allelic distribution of *AICDA* wild-type (wt) and the acceptor splice site variant (mut, c.428-1G > T) is indicated (n.a., not assessed). **b** Sequence chromatograms (gDNA) of the acceptor splice site before *AICDA* exon 4 in a healthy control (HC), the parents and both patients. Resulting nucleotide sequences are depicted below. **c** Gel electrophoresis of *AICDA* exon3/4 boundary RT-PCR products derived from CD40L/IL-21 stimulated B cells of a healthy control (HC), the affected patient (II.3) and the father (I.2). The chromatograms and resulting nucleotide sequences of the corresponding bands are depicted below. **d** Schematic representation of the *AICDA* exons and the 10 amino acid (aa) deletion in AID-ΔE4a. A DNA segment encoding a highly conserved alpha helix is indicated in darker grey [22]. Different functional AID domains are shown in the lower part [9] (NLS, nuclear localization signal; NES, nuclear export signal)

The index case (patient II.2) was the second child of consanguineous parents of Kurdish descent. She suffered from recurring otitis media necessitating multiple antibiotic treatments. She underwent adenotomy and insertion of tympanostomy tubes at the age of 8 months as well as tonsillotomy with 24 months without lasting improvement. At the time of diagnosis at the age of 4 years, she suffered from chronic otitis media and externa due to methicillin-resistant *S. aureus*. Her younger sister (patient II.3) suffered from recurrent respiratory infections, e.g., chronic serous otitis media. Tonsillotomy, adenotomy as well as insertion of tympanostomy tubes was performed at the age of 1.5 years. Re-adenotomy was done at the age of 3.5 years. Up to now, no additional signs of lymphoproliferation (e.g., splenomegaly or enlarged lymph nodes) and no overt clinical signs of autoimmune disease occurred in both patients. The elder sister (II.1) and the parents (I.1 and I.2) do not have a history of recurrent infections (Fig. 1a).

Laboratory analysis showed high IgM serum levels and absent IgG and IgA levels in both patients (Table 1). Additionally, specific antibody titers were assessed in patient II.2 and were absent despite previous vaccination (Table 1). Both patients displayed an almost complete absence of IgD^−^IgM^−^CD27^+^ switched memory B cells (Table 1). The frequency of CD19^+^CD27^−^IgD^+^IgM^+^CD24^−^CD38^+^ mature naïve B cells was reduced in both patients, whereas that of CD19^+^CD27^−^IgD^+^IgM^+^CD24^+^^+^CD38^+^ transitional B cells was increased (Table 1). The clinical, laboratory and immunological phenotype of both patients was compatible with a complete defect in CSR suggesting hyper-IgM syndrome. Subcutaneous immunoglobulin replacement therapy (IRT) was initiated in both patients. Both patients benefited from IRT and no longer suffered from recurrent and/or severe infections suggesting antibody deficiency as cause of the reported symptoms. Both parents were healthy, and immunological assessment displayed normal levels of serum immunoglobulin and specific antibody titers after vaccination (Table S1). Additionally, flow cytometric analysis of their peripheral blood B cells also did not reveal signs of disturbed CSR in both parents (Table S1). We therefore assumed that the disease is inherited as an autosomal-recessive trait in these kindred.

**Table 1:** Immunological features of the two patients

Population/Parameter	II.2	Reference values	II.3	Reference values
White blood cells (/µl)	4,900	4,800 – 13,080	16,500	5,400 – 13,800
Granulocytes (%)	61	50 – 80	54.2	25 – 68
Lymphocytes (%)	28	25 – 50	38.8	28 – 59
CD19^+^ (/µl)	277	390 – 1,400	1,061	390 – 1,400
CD27^−^/ CD19^+^ (%)	90.1	76.3—89.2	80.2	76.3—89.2
IgD^+^IgM^+^CD24^+^CD38^+^CD27^−^/ CD19^+^ (%)	41.1	50.3—67.2	30.3	50.3—67.2
IgD^+^IgM^+^CD24^+^CD38^−^CD27^−^/ CD19^+^ (%)	8.4	2.8—10.6	17	2.8—10.6
IgD^+^IgM^+^CD24^+^^+^CD38^+^^+^CD27^−^/ CD19^+^ (%)	25.4	6.5—17.1	17.4	6.5—17.1
CD27^+^/ CD19^+^ (%)	8.5	9.1—21.4	17.3	9.1—21.4
IgD^+^IgM^+^CD27^+^/ CD19^+^ (%)	6.5	3.7—9.4	14.9	3.7—9.4
IgD^−^IgM^+^ CD27^+^/ CD19 ^+^ (%)	1	0.1—0.7	0.7	0.1—0.7
IgD^+^IgM^−^CD27^+^/ CD19^+^ (%)	0.7	0.9—2.6	1.6	0.9—2.6
IgD^−^IgM^−^CD27^+^/ CD19^+^ (%)	0.3	2—8.6	0.1	2—8.6
IgD^−^IgM^−^CD27^−^/ CD19^+^ (%)	0.6	1.7—8.4	0.3	1.7—8.4
CD27^+^ ^+^CD38^+^ ^+^/ CD19^+^ (%)	0.1	0.1—0.4	0.04	0.1—0.4
CD21^−^CD38^−^/ CD19^+^ (%)	1.7	1.8—6.5	0.4	1.8—6.5
CD3^−^CD56^+^ (/µl)	120	130 – 720	503	130 – 720
CD3^+^CD4^+^ (/µl)	556	700 – 2,200	1,751	700 – 2,200
CD31^+^CD45RA^+^/ CD4^+^ (%)	44.4	53 – 74	55.1	52 – 67
CD27^+^CD45RA^+^/ CD4^+^ (%)	55.3	65 – 84	75.1	72 – 84
CD27^+^CD45RA^−^/ CD4^+^ (%)	35.2	14 – 33	21.8	15 – 26
CD27^−^CD45RA^−^/ CD4^+^ (%)	8.7	0.4 – 3.4	3.0	0.5 – 2.2
CD27^−^CD45RA^+^/ CD4^+^ (%)	0.8	0.1 – 1.1	0.1	0 – 0.9
CD3^+^CD8^+^ (/µl)	209	490 – 1,300	653	490 – 1,300
CD27^+^CD45RA^+^/ CD8^+^ (%)	57.1	55 – 91	60.6	57 – 91
CD27^+^CD45RA^−^/ CD8^+^ (%)	26.8	6 – 23	23.7	6 – 22
CD27^−^CD45RA^−^/ CD8^+^ (%)	7.5	0.4 – 13	6.4	0 – 7
CD27^−^CD45RA^+^/ CD8^+^ (%)	8.6	0.8 – 22	9.2	0.6 – 16
IgM (g/l)	7.2	0.24 – 2.1	6.3	0.52 – 1.9
IgA (g/l)	< 0.1	0.27 – 1.95	< 0.05	0.3 – 1.9
IgG (g/l)	< 0.3	5.04 – 14.64	< 0.4	5.4 – 13.4
Anti-Tetanus IgG (U/ml)	< 0.01	> 0.1	n.a	> 0.1
Anti-PcP IgG (mg/l)	< 3.3	0.8—262	n.a	0.8—262

Reference values are age-matched from the local laboratory and [23, 24] (n.a., not assessed)

### Intronic Splice Site *AICDA* Mutation Causing a 30 bp In-Frame Deletion in Exon 4

WES was performed in the index patient II.2 and identified a homozygous mutation in *AICDA* (c.428-1G > T; reported as rs766361035) at an evolutionary conserved nucleotide at the acceptor splice site of intron 3 (Fig. 1a, b). No other rare variants were detected in genes associated with hyper-IgM syndrome (*CD40, UNG, PIK3CD, PIK3R1, INO80, MSH6* and *ATM*). The mutation was absent in public databases in homozygous state and was found in one healthy individual in heterozygous state (12–8604923-C-A, gnomAD v3.1.2, minor allele frequency 1.51*10^–6^). The mutation was not previously reported in a clinical context (HGMD, PubMed, ClinVar). The mutation was confirmed by Sanger sequencing and detected in a homozygous state in both patients and a heterozygous state in both parents (Fig. 1a, b). This data suggest that the identified *AICDA* mutation segregates with the clinical and immunological phenotype in this kindred and is disease causing in a homozygous state.

To confirm the predicted impact of this mutation on *AICDA* gene splicing, we studied *AICDA* mRNA expression in B cells stimulated with CD40L and IL-21. We observed an altered splicing product after amplification of the exon 3–4 region in B cells from patient II.3 (Fig. 1c). Sequencing of this splicing product revealed a 30-bp in-frame deletion at the beginning of exon 4 compared to the dominant wild-type product observed in the healthy control B cells (Fig. 1c). This splicing product is equivalent to the known splice variant AID-ΔE4a (NCBI accession number AY536517), which has been detected in malignant B cells from patients with chronic lymphocytic leukemia but also tonsillar B cells from healthy control individuals [25]. No wild-type transcript could be detected in the patient`s B cells. The altered splicing product was present together with the wild-type transcript in the father’s B cells (Fig. 1c). Additionally, a discrete band at the size of the altered splicing product could also be detected in control B cells, suggesting that this splice product might be expressed at low levels in activated healthy control B cells. Hence, this novel *AICDA* mutation completely disrupts the splice acceptor site of intron 3 and leads to usage of an alternative splice site within exon 4, resulting in the sole expression of the truncated AID-ΔE4a variant lacking 10 highly conserved amino acids in homozygous patients (Fig. S1).

### Enhanced Germinal Center Reactions in AID-ΔE4a Patients

**Fig. 2:**
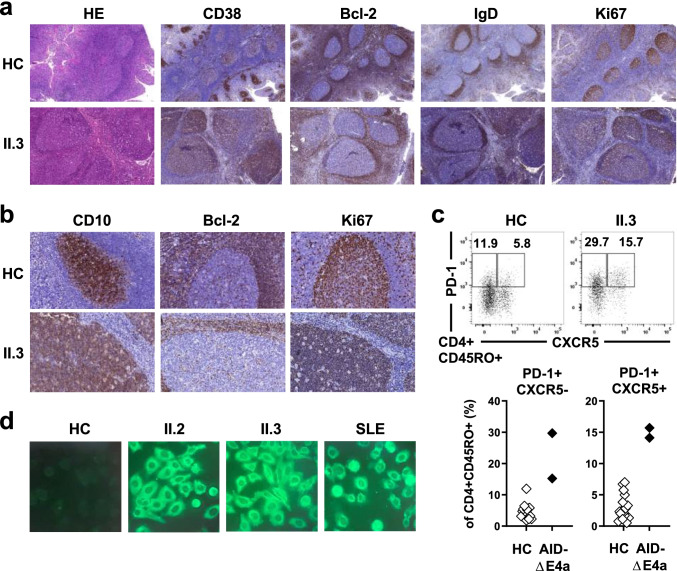
Exaggerated germinal center activity in AID-ΔE4a patients. **a** Representative histological (HE staining) and immunohistological (CD38, Bcl-2, IgD, Ki-67) analysis of tonsil tissue derived from patient II.3 and a control individual (magnification × 100). **b** Immunohistological analysis (CD10, Bcl-2 and Ki67) of germinal centers (magnification × 400). **c** Representative dot plot of PD-1 and CXCR5 surface expression on peripheral blood CD4^+^ CD45RO^+^ T cells (upper part) and frequencies of T_FH_ (PD-1^+^CXCR5^+^CD45RO^+^CD4^+^) and T_PH_ (PD-1^+^ CXCR5^−^CD45RO^+^CD4^+^) cells in AID-ΔE4a patients and age-matched healthy controls as assessed by flow cytometry (lower part). **d** Indirect immunofluorescence staining of HEp-2 cells using FITC-labeled secondary anti-IgM-antibodies with indicated sera derived from both AID-ΔE4a patients, a SLE patient and a healthy control (dilution 1:160)

Enlargement of GCs has been reported in AID-deficiency in both mice and humans [26]. Both patients underwent surgery for adenoid and tonsillar hypertrophy offering insights into disease processes within secondary lymphatic tissues. Remarkably, massive follicular hyperplasia was present in adenoid and/or tonsil tissues in both patients. Indeed, in comparison with control tissues obtained from patients without primary immunodeficiencies, GCs were 4 to 10 times larger in the AID-ΔE4a patients (Fig. 2a, b and Fig. S2). The interfollicular areas and mantle zones were preserved with mantle zone B cells showing a regular phenotype (CD23^+^CD38^−^Bcl-2^+^IgM^+^IgD^+^Ki67^−^) (Fig. 2a). The follicular dendritic network appeared regular within giant GCs. However, IgD was expressed in many GC B cells in contrast to controls in which IgD^+^ GC B cells are only found scattered in small numbers. GC B cells were surrounded by a dense network of tangible body macrophages causing a typical starry sky pattern of giant GCs and suggesting increased cellular turnover within GC of AID-ΔE4a patients as previously described in other AR-AID-deficient patients [1]. In line with this observation, enlarged and disordered GCs correlated with increased frequencies of activated, circulating follicular helper (T_FH_, PD-1^+^ CXCR5^+^CD4^+^) but also peripheral helper (T_PH_, PD-1^+^ CXCR5^−^CD4^+^) T cells present in peripheral blood of both patients (Fig. 2C). In addition, serum analysis from AID-ΔE4a patients revealed IgM autoantibodies that reacted against cell structures of HEp-2 cells, similar to autoreactive IgM autoantibodies from SLE patients, suggesting a breach in B cell tolerance in these patients (Fig. 2d).

Remarkably, clear reduction of GC size was observed in histological analysis of adenoid vegetation of patient II.3 15 months after initiation of IRT (Supplementary Fig. S3a). In parallel, the frequencies of activated, circulating T_FH_ and T_PH_ cell normalized following IRT in both patients (Fig. S3b). Hence, similar to other patients with AR AID-deficiency, exaggerated GC activity is also present in AID-ΔE4a patients and may result from a failure in producing high-affinity, class-switched antibodies that may be partially corrected by IRT.

### Complete Defect in CSR but Preserved SHM in AID-ΔE4a Patients is Reminiscent of AD-AID Deficiency

**Fig. 3:**
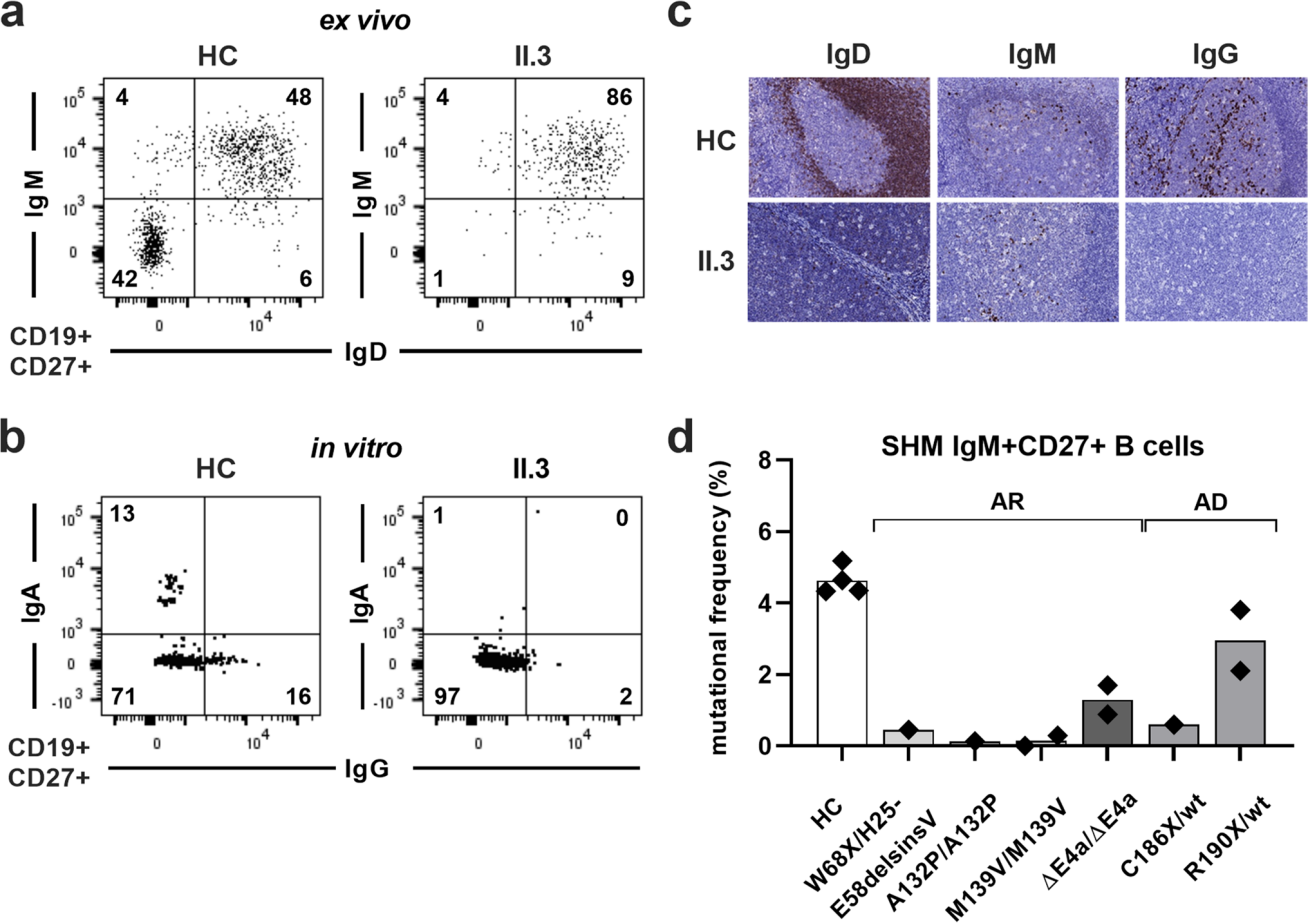
Defective class switch recombination and disturbed somatic hypermutation in AID-ΔE4a patients. **a** Representative dot plots of surface IgM and IgD expression on peripheral blood CD19^+^CD27^+^B cells from patient II.3 and an age-matched healthy control (HC). **b** Representative dot plots of surface IgA and IgG staining on sorted peripheral blood CD19^+^CD27^+ ^B cells from patient II.3 and an age-matched healthy control (HC) after *in vitro* stimulation with CD40L and IL-21. **c** Immunohistological analysis (IgD, IgM and IgG) of tonsil tissues derived from patient II.3 and a control individual (magnification × 400). **d** Mutational frequency of the variable region of the immunoglobulin heavy chain of sorted non-switched memory B cells (CD19^+^CD27^+^ gM^+^Ig^+^) in genetically different groups of HIGM2 patients; each symbol represents one individual, bars represent the mean mutational frequency within distinct patient groups (AR, autosomal-recessive; AD, autosomal-dominant)

The pattern with enhanced GC reaction and increased T_FH_ cell production resembles that of other AR-AID patients lacking CSR and SHM activity [16]. Indeed, both AID-ΔE4a patients displayed signs of a complete defect in CSR *in vivo,* which was recapitulated in CD40L/IL-21-stimulated naïve B cells *in vitro* from patient II.3 (Fig. 3a, b and Table 1). No IgG-expressing plasma cells could be detected within T cell areas or GCs, which underlines the complete defect of CSR in AID-ΔE4a patients (Fig. 3c and Fig. S2). However, previous reports suggested that the AID-ΔE4a variant differentially affects CSR and SHM function *in vitro,* rendering SHM active, a finding that is currently restricted to AD-AID patients with C-terminal *AICDA* mutations [25, 27, 28]. We therefore assessed the functional impact of the AID-ΔE4a variant on SHM by comparing the mutational frequency within the variable region of the IgH genes amplified from CD19^+^CD27^+^IgM^+^ NSM B cells isolated from AID-ΔE4a patients to counterparts from patients with other *AICDA* mutations and healthy controls. Whereas the mutation frequency in AID-ΔE4a patients was lower than in healthy control individuals, we still could detect a considerable number of mutated nucleotides within the IgH variable regions of their NSM B cells (Fig. 3d). The mutation frequency in AID-ΔE4a patients was higher than in other AR-AID-deficient patients with distinct *AICDA* mutations that completely abrogate AID expression and/or function (Fig. 3d). Furthermore, the extent of SHM in the AID-ΔE4a patients appeared within the range of SHM frequencies in AD-AID patients harboring heterozygous C-terminal *AICDA* mutations (Fig. 3d and Table S2). Hence, the AID-ΔE4a variant resembles C-terminal truncated AID variants in that it completely impairs CSR but preserves some SHM activity.

### Impaired SHM Targeting Distinguishes AID-ΔE4a from AD-AID Patients

SHM but not CSR controls GC reactions [16, 29]. Since both AD-AID and AID-ΔE4a patients display partially functional SHM processes but differ in their GC responses that appears normal in AD-AID but hyperplastic in AID-ΔE4a patients, we speculated that qualitative differences in SHM rather than the amount of SHM might account for this discrepancy. We therefore compared SHM within NSM B cells between the two AID-ΔE4a patients and three AD-AID patients. We found that the frequency of mutated sequences did not differ significantly between AD-AID and AID-ΔE4a patients (69.4% and 58.0%, respectively, p = 0.24, Table S2 and S3). Mutation frequency within mutated sequences was only modestly decreased in AID-ΔE4a compared to AD-AID patients (3.1 ± 2.9% and 1.9 ± 1.4%; p = 0.05).

**Fig. 4:**
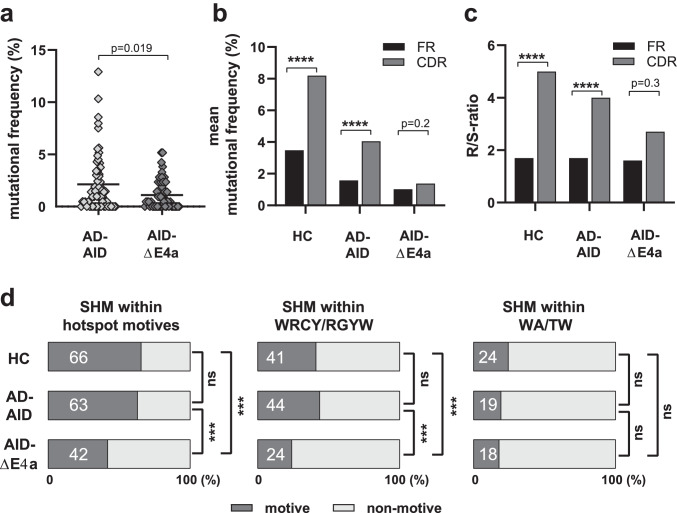
Defective targeting of somatic hypermutation in AID-ΔE4a patients. **a** Mutational frequency in the variable region of the immunoglobulin heavy chain of sorted non-switched memory B cells (CD19^+^CD27^+^IgM^+^) from AD-AID and AID-ΔE4a patients; each symbol represents one sequence, horizontal lines indicate the mean; p-value as computed by two-tailed, unpaired Student’s t-test. **b** Mean mutational frequency of framework and complementarity-determining regions (FR and CDR, respectively) within indicated group of patients and healthy controls (HC). (Statistical significances as determined by Chi-square test with Yates’ correction are indicated, ****, *p* < 0.0001). **c** Ratios of replacement (R) versus silent mutations (S) in framework and complementarity determining-regions. (Statistical significances as determined by Fisher’s exact t test; ***, *p* < 0.001; ****, *p* < 0.0001). **d** Frequency of mutations located in indicated hotspot motives out of all mutations in healthy controls (HC), AD-AID and AID-ΔE4a patients (on the left: mutations in either WRCY/RGYW or WA/TW). (Statistical significances as determined by Fisher’s exact t test between the two indicated groups; ***, *p* < 0.001; ns, not significant)

We then assessed the localization of SHM within IgH V regions. Mutations were significantly enriched in the complementarity-determining regions (CDRs) compared to framework regions (FWRs) of IgH genes from healthy control individuals with higher replacement/silent (R/S) ratios in CDRs than FWRs (Fig. 4b, c). Similar SHM enrichment in IgH CDR3s and elevated R/S ratios indicative of effective affinity maturation processes were also observed in AD-AID patients and are associated with proper GC downregulation (Fig. 4b, c). In contrast, SHM were distributed almost evenly among CDRs and FWRs and R/S-ratios were not significantly increased in CDRs compared to those in FWRs in AID-ΔE4a patients (Fig. 4b, c and Table S3). We conclude that SHM targeting is specifically impaired in patients expressing the AID-ΔE4a variant.

To further test this hypothesis, we assessed the distributions of mutations within known SHM hotspot motives [5]. We found that 66% and 63% of SHM were identified in hotspot motives in IgH sequences from healthy controls and AD-AID patients, whereas only 42% of SHM were targeted to hotspot motives in AID-ΔE4a patients (Fig. 4d). Decreased SHM targeting in AID-ΔE4a patients resulted from the decreased accumulation of SHM at WRCY/RGYW hotspot motives (Fig. 4d). Of note, the frequency of WRCY/RGYW motives within the analyzed V_H_ gene segments was not lower in AID-ΔE4a patients compared to healthy controls and cannot account for the reduced mutational targeting of these hotspot motives (Fig. S4 and Table S3). In contrast, AID-ΔE4a and AD-AID patients did not display lower SHM frequencies at WA/TW hotspot motives, which are targeted by POLH and arise at A and T bases surrounding the original U:G mismatch (Fig. 4d) [6]. Hence, mutational targeting of CDRs and AID hotspot motives WRCY/RGYW is selectively impaired in the patients expressing the AID-ΔE4a variant but not in AD-AID patients who express C-terminal truncated AID variants.

Several distinct pathways operate on resolving uracil lesions created by AID and thereby further shape the mutational pattern of SHM (Fig. S5a) [6]. We therefore aimed at inferring the mutational consequences of lesions created within the IgH genes. There was no difference regarding the particular type of base pairs, which were targeted for mutations in AID-ΔE4a patients compared to healthy controls; however, transitions and transversions at C and T were increased and mutations at G decreased in AID-ΔE4a patients (Fig. S5b). Differing from that, mutations were enriched at G/C base pairs in AD-AID patients and this preference was accounted for by transitions and transversions at C and transversions at G (Fig. S5b, c and Table S3). Finally, a stepwise reduction of the A over T ratio could be detected from healthy controls (1.7) over AD-AID patients (1.2) to AID-ΔE4a patients (0.7) implying a shift of the strand bias towards error-prone synthesis at the transcribed strand in the latter group (Fig S5d and Table S3).

## Discussion

Herein we report the functional impact of a novel *AICDA* intronic splice site mutation identified in two AR-AID patients on CSR, SHM and GC responses. This *AICDA* mutation disrupts the splice acceptor site of exon 4 and results in the sole expression of a truncated AID variant (AID-ΔE4a) when present in a homozygous state. This new AID variant abrogated CSR but only partially affected SHM as previously observed in AD-AID patients [10]. However, unlike AD-AID deficiency, AID-ΔE4a patients displayed enhanced GC responses and secreted autoantibodies previously associated with the complete loss of SHM activity [16, 29]. Interestingly, AID-ΔE4a but not AD-AID patients revealed impaired targeting of mutational hotspot motives and distorted mutational patterns, suggesting that qualitative alterations rather than merely reduced enzymatic activity account for the impaired SHM process in AID-ΔE4a patients.

The AID-ΔE4a variant has already been detected as an alternative splicing product in tonsil B cells from healthy donors as well as CLL B cells [25]. There are apparent discrepancies regarding the functional outcome of the AID-ΔE4a variant [25, 27, 28]. Conflicting results between different experimental *in vitro* systems and *in vivo* analyses were also obtained when assessing the enzymatic function of other AID variants [9]. The AID-ΔE4a splice variant has been reported to display proper subcellular localization but a pGFP* reversion assay in murine 70Z/3 cells suggested increased SHM activity [25]. In contrast, van Maldegem et al*.,* using the same assay in NIH-3T3 cells, argued for a technical artifact, which seems to elicit this phenomenon [25, 27, 28]. Furthermore, they demonstrated that AID-ΔE4a was not able to deaminate cytidine in an oligonucleotide substrate *in vitro* and therefore should be regarded as catalytically inactive [27, 28]. The analysis of AID-ΔE4a patients reveals that SHM is only partially affected by this variant *in vivo* but present compared to patients expressing AID loss-of-function variants. The amount of SHM associated with the AID-ΔE4a variant was in the range of AD-AID patients known to possess residual SHM activity [10, 16].

In contrast to AR-AID patients with *AICDA* mutations that directly impinge on cytidine deaminase activity or even abolish AID protein expression and who suffer from defective SHM and CSR [15], both functional *in vitro* studies and our *in vivo* analysis of two HIGM2 patients, who solely expressed the AID-ΔE4a variant, revealed the inability of this AID splicing product to catalyze CSR [25]. Beside the absence of class-switched memory B cells, both patients also showed alterations within their naïve B cell compartment with increased frequencies of transitional B cells. Increasing evidence from mouse models and human studies indicated that AID is also expressed in developing B cells of the bone marrow and functions in central B cell tolerance [18, 19, 30, 31]. Hence, the alterations within the transitional B cell compartment could be a result of defective AID-function during B cell development in the bone marrow. Alternatively, elevated BAFF levels that are present in AID-deficient patients might augment the generation of transitional B cells [16].

Defective CSR with residual SHM activity is a characteristic feature of AD-AID patients harboring heterozygous *AICDA* mutations, which affect the last 8–12 amino acid C-terminal nuclear export signal domain encoded by exon 5 [10]. The AID-ΔE4a variant does not affect this C-terminal AID domain but lacks a sequence of 10 highly conserved amino acids encoded at the beginning of exon 4, which does not directly affect the cytidine deaminase domain. Hence, conformational alterations that indirectly entail decreased deaminase activity and/or impaired cytoplasmatic-nuclear shuttling might account for the impaired function of the AID-ΔE4a variant. However, the observation that CSR and SHM are differentially affected and mutational targeting and the mutational pattern are selectively distorted—as seen by lost preference for CDRs, reduced targeting of WRCY/RGYW but not WA/TW hotspots and altered strand targeting—may argue against these considerations. Indeed, these observations may point to a selective qualitative targeting defect of AID rather than a globally decreased enzymatic activity of the AID-ΔE4a variant. Several co-factors have been described, which are essential for proper AID function and impaired interaction with these co-factors has been suggested to account for impaired function of known AID variants [11, 13, 14, 32]. Indeed, the dominant negative effect of the C-terminal AID variants in AD-AID patients is ascribed to depletion of CSR-specific co-factors from the wild-type allele [13]. Furthermore, CTNNBL1 interacts with AID, thereby facilitating AID shuttling to the nucleus. Mutations in both CTNNBL1 and AID have been described to interfere with this interaction and impair CSR and SHM in humans [33]. In line with this, the ten amino acids missing in the AID-ΔE4a variant form a highly conserved alpha helix, which seems to be essential for interaction with other AID co-factors. In detail, the RNA-binding protein ROD1 (*PTBP3*) serves as a guiding system for AID and is required for AID-targeting to immunoglobulin loci [22]. ROD1 interacts with AID via a highly conserved loop, comprising the amino acid residues 140–151 [22]. Intriguingly, the amino acid residues 143–152 lacking in the AID-ΔE4a variant exactly match the essential ROD1 interaction site. Hence, the abolished CSR and distorted SHM pattern observed in the two HIGM2 patients expressing the AID-ΔE4a variant may result from the inability of their truncated AID to bind ROD1. We did not find any other potential disease causing mutations in known hyper-IgM-syndrome-related genes. However, we cannot completely rule out the presence of other noncoding variants or small copy number variants that might have been missed by whole-exome sequencing and additionally modify the immunological phenotype of these patients.

AR-AID patients who lack SHM secreted IgM autoantibodies associated with increased T_FH_ cells, whereas AD-AID or UNG-deficient patients with preserved SHM activity did not display autoantibodies or elevated T_FH_ frequencies [16]. This suggested that SHM but not CSR is essential for maintaining B cell tolerance in humans [16]. Additionally, SHM but not CSR is also involved in controlling GC reactions since mice harboring distinct *AICDA* mutations that abrogate SHM but not CSR develop GC hyperplasia [29]. It was, therefore, not expected to observe increased GC activity and IgM autoantibodies in the AID-ΔE4a patients whose ability to exert SHM is still preserved with SHM frequencies almost similar to AD-AID patients. Several explanations may account for this observation: first, the slightly lower frequency of SHM in the AID-ΔE4a patients compared to AD-AID patients could provoke follicular hyperplasia and/or autoimmunity. Second, the defective targeting of SHM to hotspot motives in AID-ΔE4a patients may result in B cell receptors (BCRs) that are less effective in binding and clearing antigens from follicular dendritic cells in GCs [17]. SHM processes that have been suggested to redeem autoreactive BCRs encoded by distinct V gene segments with inherent self-reactivity as well as defects in SHM targeting might also impair reversion of self-reactivity and activation of these autoreactive B cells [34]. Remarkably, giant GC as well as increased T_FH_ and T_PH_ cells resolved in the AID-ΔE4a patients after initiation of IRT, suggesting that provision of correctly mutated antibodies besides preventing severe infections may also dampen immune dysregulation in these patients.

## Conclusion

In summary, we have described the clinical phenotype and functional outcome of two HIGM2 patients harboring a novel homozygous *AICDA* splice site mutation that leads to the sole expression of the AID-ΔE4a variant. Both patients lacked CSR but still possessed residual SHM activity. Increased GC activity and secretion of IgM autoantibodies were reminiscent of AR-AID patients that completely lack SHM processes but might be explained by impaired SHM targeting to hotspot motives induced by the AID-ΔE4a variant. Hence, the level of enzymatic activity as well as precise AID targeting potentially mediated by AID co-factors appears to influence the phenotype of AID-deficiencies.

## Supplementary Information

Below is the link to the electronic supplementary material.

Supplementary file1 (PDF 969 KB)

## Data Availability

Data are available from the corresponding author upon reasonable request.
