# Supplementary material for: A Novel AICDA Splice-Site Mutation in Two Siblings with HIGM2 Permits Somatic Hypermutation but Abrogates Mutational Targeting
Source: J Clin Immunol. 2022 Mar 5;42(4):771–82. doi: 10.1007/s10875-022-01233-5 (PMC9166864; doi:10.1007/s10875-022-01233-5)
Supplement: Supplementary file 1 — Supplementary file1 (PDF 969 KB) [file 10875_2022_1233_MOESM1_ESM.pdf]

A novel *AICDA* splice-site mutation in two siblings with HIGM2 permits somatic hypermutation but abrogates mutational targeting

Supplemental Data

Supplemental Tables

Table S1 - Immunological features of the two parents. Reference values are age matched from the local laboratory and [1, 2].

Population/ Parameter	I.2	I.1	Reference values
White blood cells (/µl)	5,710	5,870	4,800 – 10,800
Granulocytes (%)	56.8	63.6	40 – 74
Lymphocytes (%)	31.5	27.2	19 – 48
CD19+ (/µl)	277	186	70 – 480
CD27-/CD19+ (%)	**90**	**86.2**	63.4 – 82.7
IgD+IgM+CD24+CD38+CD27-/ CD19+ (%)	64.9	64.3	40.1 – 65.4
IgD+IgM+CD24+CD38-CD27-/ CD19+ (%)	**2.5**	**4.1**	4.9 – 10.3
IgD+IgM+CD24++CD38++CD27-/ CD19+ (%)	2.8	3.7	2.1 – 7.6
CD27+/CD19+ (%)	**8.9**	**12.4**	15.3 – 34.3
IgD+IgM+CD27+/CD19+ (%)	**1**	**3**	6 – 13.8
IgD-IgM+CD27+/CD19+ (%)	**0.2**	**0.2**	0.5 – 1.8
IgD+IgM-CD27+/CD19+ (%)	**0.3**	**0.4**	0.9 – 4.9
IgD-IgM-CD27+/CD19+ (%)	7.5	8.9	4.1 – 17.7
IgD-IgM-CD27-/CD19+ (%)	**17**	**11.2**	2.7 – 8.6
CD27++CD38++/CD19+ (%)	**0**	0.1	0.1 – 1.5
CD21-CD38-/CD19+ (%)	**15.6**	**12.9**	2.2 – 7.5
CD3-CD56+ (/µl)	200	**84**	110 – 570
CD3+CD4+ (/µl)	631	809	530 – 1300
CD3+CD4+CD45RO+ (/µl)	357	482	216 – 490
CD3+CD8+ (/µl)	354	**272**	330 – 920
CD3+CD8+CD45RO+ (/µl)	168	146	33 – 189
IgM (g/l)	1.06	1.55	0.4 – 2.4
IgA (g/l)	1.51	1.93	0.7 – 3.7
IgG (g/l)	15.24	11.21	6.9 – 16
Anti-Tetanus IgG (IU/ml)	4.42	0.28	> 0.1

Table S2 – Mutational frequency

Frequency of mutated immunoglobulin heavy chain sequences and mutational frequency in non-switched memory B cells of healthy controls, AR-AID patients and AID- ΔE4a patients. Two-tailed p-values determined by either ° Fisher's exact t test between the two indicated groups or ‡ Chi square with Yates' correction between the two indicated groups.

	HC	AR-AID	ΔE4a-AID	p (HC vs AR-AID)	p (HC vs ΔE4a - AID)	p (ΔE4a vs AR-AID)
Mutated sequences/ Analyzed sequences (%)	52/54 (96.3)	14/54 (25.9)	29/50 (58.0)	0.0002°	<0.0001°	0.0013°
Mutated nucleotides/ Analyzed nucleotides (%)	524/11511 (4.6)	25/11381 (0.2)	117/10647 (1.1)	<0.0001‡	<0.0001‡	<0.0001‡

Table S3 – Pattern of Somatic Hypermutation

Mutational characteristics of the immunoglobulin heavy chain sequences. Two-tailed p-values determined by either ° Fisher's exact t test between the two indicated groups, ‡ Chi square with Yates' correction between the two indicated groups; § Fisher's exact t test FR vs. CDR or † Chi square with Yates' correction FR vs. CDR within the same group.

	HC	AD-AID	ΔE4a-AID	p (HC vs AD-AID)	p (HC vs ΔE4a - AID)	p (ΔE4a vs AD-AID)
Mutated sequences/ analyzed sequences (%)	52/54 (96.3)	43/62 (69.4)	29/50 (58.0)	0.0002°	<0.0001°	0.24°
Mutated nucleotides/ analyzed nucleotides (%)	524/11511 (4.6)	275/12978 (2.1)	117/10647 (1.1)	<0.0001‡	<0.0001‡	<0.0001‡
Transitions/ total mutations (%)	308/524 (58.8)	156/275 (56.7)	73/117 (62.4)	0.6°	0.53°	0.32°
Transitions at G/C / total mutations at G/C (%)	172/292 (58.9)	104/191 (54.5)	36/63 (57.1)	0.35°	0.89°	0.77°
Mutations at G/C / total mutations (%)	292/524 (55.7)	191/275 (69.5)	63/117 (53.8)	0.0002°	0.76°	0.0038°
Transitions at A/T / total mutations at A/T (%)	136/232 (58.6)	52/84 (61.9)	37/54 (68.5)	0.7°	0.22°	0.47°
Mutations at A/T / total mutations (%)	232/524 (44.3)	84/275 (30.5)	54/117 (46.2)	0.0002°	0.76°	0.0038°
Replacement/silent mutations in FR (ratio)	196/113 (1.7)	100/60 (1.7)	52/32 (1.6)	<0.0001§	0.0021§	0.29§
Replacement/ silent mutations in CDR (ratio)	179/36 (5.0)	92/23 (4.0)	24/9 (2.7)			
Mutations in FR/ nucleotides in FR (%)	309/8885 (3.5)	160/10139 (1.6)	84/8250 (1.0)	<0.0001†	<0.0001†	0.17†
Mutations in CDR/ nucleotides in CDR (%)	215/2626 (8.2)	115/2839 (4.1)	33/2397 (1.4)			
Mutations in RGYW or WRCY/ total mutations (%)	215/524 (41)	120/275 (43.6)	28/117 (23.9)	0.5°	0.0005°	0.0003°
Mutations in WA or TW/ total mutations (%)	128/524 (24.4)	53/275 (19.3)	21/117 (17.9)	0.15°	0.15°	0.66°
Mutations in hotspot motives/ total mutations (%)	343/524 (65.5)	173/275 (62.9)	49/117 (41.9)	0.48°	0.0001°	0.0001°
Adenins (A) in analyzed sequences/ analyzed nucleotides (%)	3004/11511 (26.1)	3274/12978 (25.2)	2834/10647 (26.6)	0.12‡	0.39‡	0.016‡
Cytosins (C) in analyzed sequences/ analyzed nucleotides (%)	2862/11511 (24.9)	3280/12978 (25.3)	2635/10647 (24.7)	0.47‡	0.86‡	0.36‡
Thymins (T) in analyzed sequences/ analyzed nucleotides (%)	2407/11511 (20.9)	2823/12978 (21.8)	2147/10647 (20.2)	0.11‡	0.18	0.0031
Guanins (G) in analyzed sequences/ analyzed nucleotides (%)	3238/11511 (28.1)	3601/12978 (27.7)	3031/10647 (28.5)	0.51‡	0.59‡	0.23‡
Nucleotides in WA/ analyzed nucleotides (%)	2504/11511 (21.8)	2754/12978 (21.2)	2146/10647 (20.2)	0.32‡	0.0037‡	0.046‡
Nucleotides in TW/ analyzed nucleotides (%)	1964/11511 (17.1)	2380/12978 (18.3)	1768/10647 (16.6)	0.0095‡	0.37‡	0.0005‡
Nucleotides in RGYW/ analyzed nucleotides (%)	2568/11511 (22.3)	2912/12978 (22.4)	2424/10647 (22.8)	0.82‡	0.42‡	0.56‡
Nucleotides in WRCY/ analyzed nucleotides (%)	2132/11511 (18.5)	2588/12978 (19.9)	1900/10647 (17.8)	0.0052‡	0.20‡	<0.0001‡

Supplemental Figures

Figure S1

Accession number	Species		Aminoc Acid Sequence	
NP_065712.1	H.sapiens	130	HRAGVQIAIMTFKDYFYCWNTFVENHERTFK	160
NP_001065277.1	P.troglodytes	130	HRAGVQIAIMTFKDYFYCWNTFVENHERTFK	160
XP_001113641.1	M.mulatta	130	HRAGVQIAIMTFKDYFYCWNTFVENRERTFK	160
NP_001003380.1	C.lupus	130	HRAGVQIAIMTFKDYFYCWNTFVENREKTFK	160
NP_001033771.1	B.taurus	131	HRAGVQIAIMTFKDYFYCWNTFVENHERTFK	161
NP_033775.1	M.musculus	130	HRAGVQIGIMTFKDYFYCWNTFVENRERTFK	160
NP_001094249.1	R.norvegicus	130	HRAGVQIGIMTFKDYFYCWNTFVENHERTFK	160
NP_001230151.1	G.gallus	130	HRAGAQIAIMTFKDFFYCWNTFVENREKTFK	160
NP_001008403.1	D.rerio	143	KRAGVQISVMTYKDFFYCWQTFVARRERSFK	173
XP_002941248.1	X.tropicalis	133	QKAGVRLAVMSYKDYFYCWNTFVESRERRFE	163
			a-helix	

Figure S1- The 10 amino acid deletion in AID-ΔE4a affects a highly conserved region

AID amino acid sequences from different species were aligned. The 10 amino acid deletion in AID-ΔE4a is highlighted in red. The grey box indicates a highly conserved alpha helix structure.

Figure S2

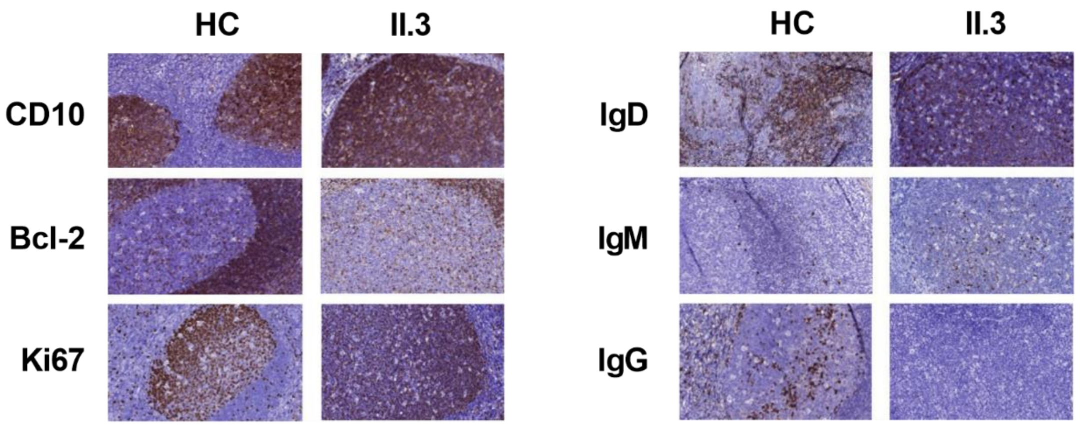

Figure S2 - Exaggerated germinal center activity in AID-ΔE4a patients

Immunohistological analysis (IgD, IgM, IgG, CD10, Bcl-2 and Ki67) of germinal centers in adenoid tissue derived from patients II.3 and the control individual (magnification x400).

Figure S3

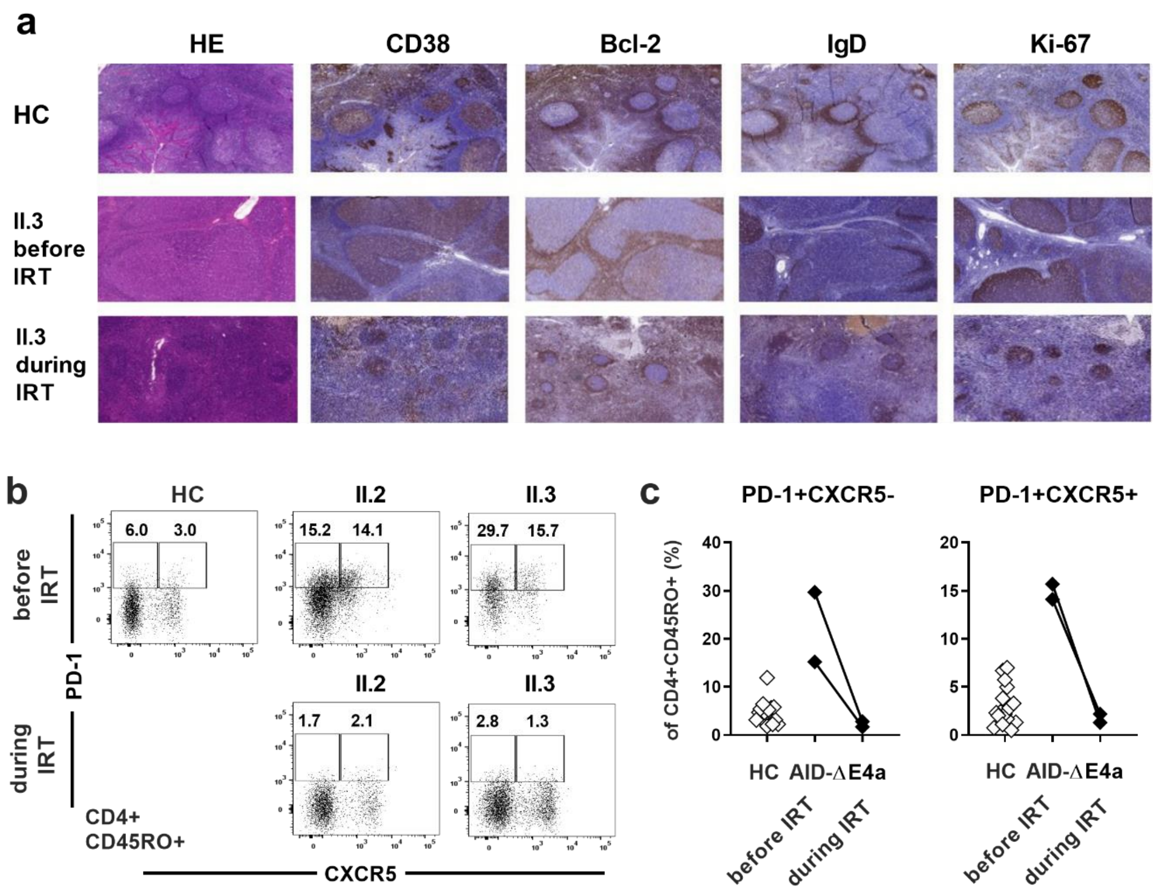

Figure S3 - Exaggerated germinal center activity in AID-ΔE4a patients resolves after initiation of IRT

a Representative histological (HE staining) and immunohistological (CD38, IgD, Bcl-2, Ki-67) analysis of adenoids derived from patient II.3 before (age 1.4 years) and after initiation of an immunoglobulin replacement therapy (IRT; age 3.5 years) as well as a control individual (magnification x100).

b Representative dot plot of PD-1 and CXCR5 surface expression on peripheral blood CD4+CD45RO+ T cells.

c Frequencies of peripheral blood T_{FH} (PD-1+CXCR5+CD45RO+CD4+) and T_{PH} (PD-1+CXCR5-CD45RO+CD4+) cells in AID-ΔE4a patients before and during IRT and age matched healthy controls as assessed by flow cytometry.

Figure S4

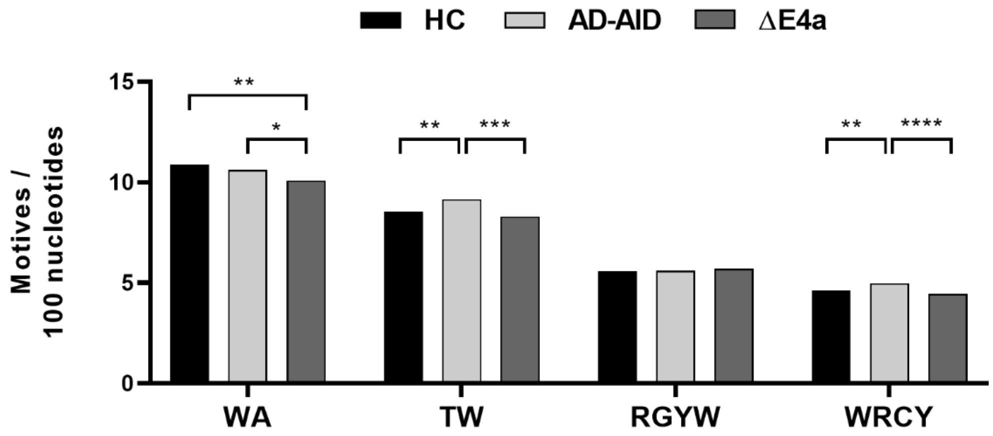

Figure S4 - Hot spot motives in AD-AID and AID- ΔE4a patients

Numbers of indicated hotspot motives per 100 analyzed nucleotides (Two-tailed p-values determined for nucleotides in indicated regions by Chi square with Yates' correction between the two indicated groups; *, p<0.05; **, p<0.01; ***, p<0.001; ****, p<0.0001).

Figure S5

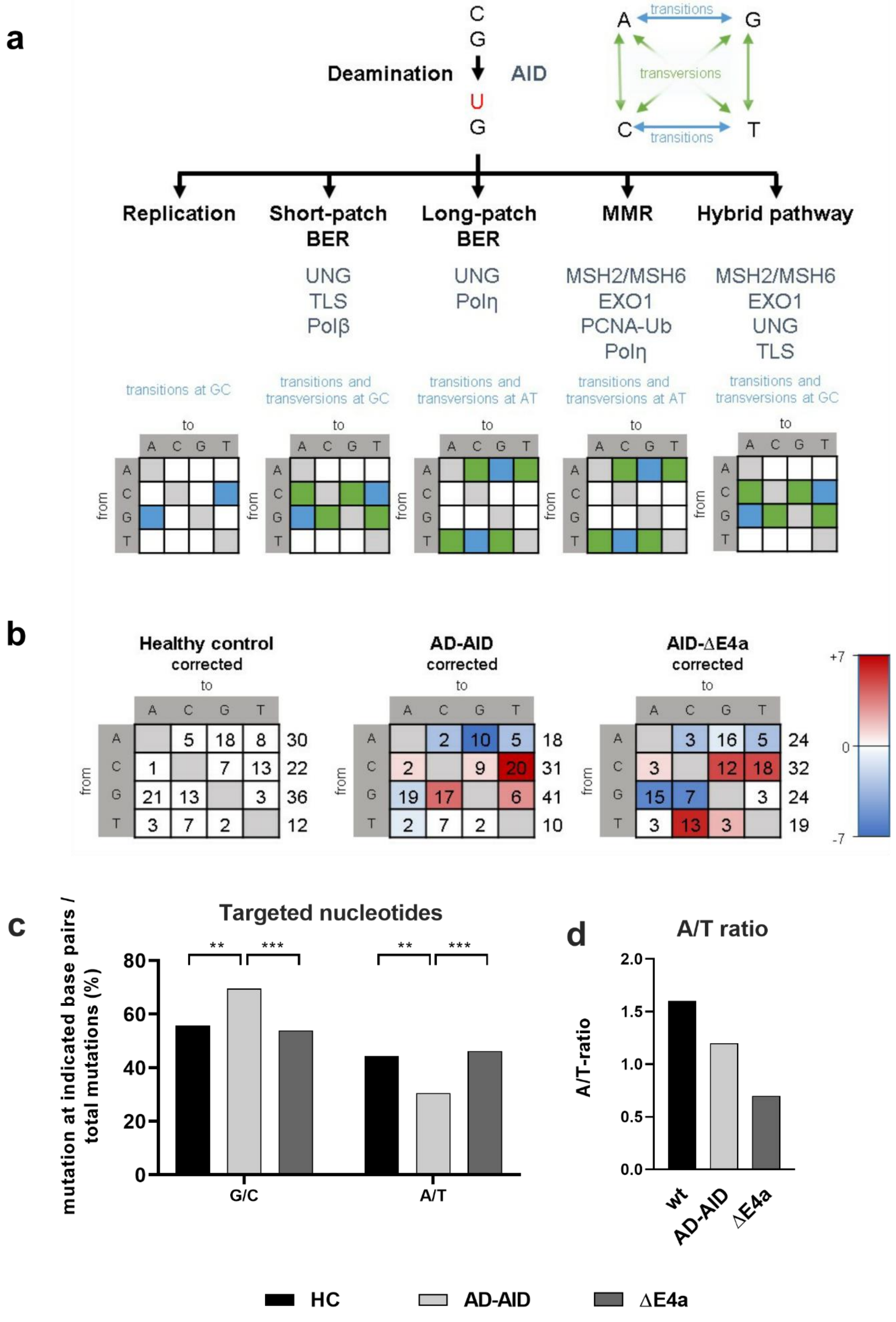
a
C
G
Deamination
AID
U
G
A
G
C
T
transitions
transversions
transitions
Replication
Short-patch BER
Long-patch BER
MMR
Hybrid pathway
UNG
TLS
Polβ
UNG
Polη
MSH2/MSH6
EXO1
PCNA-Ub
Polη
MSH2/MSH6
EXO1
UNG
TLS
transitions at GC
transitions and transversions at GC
transitions and transversions at AT
transitions and transversions at AT
transitions and transversions at GC
to
from
A C G T
b
Healthy control corrected
AD-AID corrected
AID-ΔE4a corrected
to
from
A C G T
+7
0
-7
c
Targeted nucleotides
mutation at indicated base pairs / total mutations (%)
0
20
40
60
80
G/C
A/T
**
***
d
A/T ratio
A/T-ratio
0.0
0.5
1.0
1.5
2.0
wt
AD-AID
ΔE4a
HC
AD-AID
ΔE4a

Figure S5 - Pathways of somatic hypermutation and alterations of mutational patterns in AD-AID and AID-ΔE4a patients

a Schematic model of somatic hypermutation pathways and involved proteins following deamination of dC by AID. Resulting base exchanges are indicated in blue for transitions and green for transversions in the tables below (adapted and modified from [3]).

b Frequency of indicated mutations out of all mutations in immunoglobulin heavy chain sequences derived from non-class switched memory B cells of healthy controls and patients, corrected for the relative number of analyzed nucleotides. For patients, the absolute difference of frequencies compared to the healthy controls is indicated by color as depicted on the right.

c Mutational frequency at indicated base pairs out of all mutations in immunoglobulin heavy chain sequences derived from non-switched memory B cells of healthy controls (HC), AD-AID as well as AID-ΔE4a patients. (Two-tailed p-values determined by Fisher's exact t test between the two indicated groups; **, $p<0.01$; ***, $p<0.001$)

d Corrected A over T ratio in the analyzed sequences of healthy controls (HC), AD-AID as well as AID-ΔE4a patients.

References

1. van Gent, R., et al., *Refined characterization and reference values of the pediatric T- and B-cell compartments.* Clin Immunol, 2009. **133**(1): p. 95-107.
2. Morbach, H., et al., *Reference values for B cell subpopulations from infancy to adulthood.* Clin Exp Immunol, 2010. **162**(2): p. 271-9.
3. IJSpeert, H., et al., *Repertoire Sequencing of B Cells Elucidates the Role of UNG and Mismatch Repair Proteins in Somatic Hypermutation in Humans.* Front Immunol, 2019. **10**: p. 1913.
